# Successful management of prolonged abdominal pregnancy in low-resource setting: a case report

**DOI:** 10.1093/jscr/rjae210

**Published:** 2024-04-02

**Authors:** Cátia Samajo Zita, Gonzalo Gonzáles Villa, Eduardo Matediana, Pita Tomás, Damiano Pizzol, Lee Smith

**Affiliations:** Department of Obstetrics and Gynecology, Central Hospital of Beira, 1363 Beira, Mozambique; Department of Obstetrics and Gynecology, Central Hospital of Beira, 1363 Beira, Mozambique; Department of Obstetrics and Gynecology, Central Hospital of Beira, 1363 Beira, Mozambique; Department of Obstetrics and Gynecology, Central Hospital of Beira, 1363 Beira, Mozambique; Operational Research Unit, Doctors with Africa CUAMM, 1363 Beira, Mozambique; Centre for Health, Performance and Wellbeing, Anglia Ruskin University, CB1 1PT Cambridge, United Kingdom

**Keywords:** prolonged pregnancy, ectopic pregnancy, low-resource setting, case report

## Abstract

Ectopic pregnancy is a life-threatening complication of pregnancy and represents the leading cause of maternal mortality in the first trimester. In developing countries early diagnosis, necessary for favorable outcomes, is often unavailable and women are often not aware of possible conditions and associated complications. Moreover, access to sexual and reproductive health services and antenatal care are limited in such settings. Finally, management options are restricted and often performed in emergency with higher risk of complications and mortality. We report here a 33-year-old woman presenting a 41 weeks abdominal pregnancy successfully managed in a low-resource setting.

## Introduction

Ectopic pregnancy (EP) is a complication of pregnancy where the embryo implants outside the uterine cavity, mainly in the Fallopian tube but also in the cervix, ovaries, and abdomen [1]. EP is life-threatening for the mother especially due to the possible consequent internal hemorrhage and it represents the leading cause of maternal mortality in the first trimester, with an estimated incidence of 5–10% of all pregnancy-related deaths [1]. Reliable epidemiological data are available only in developed countries with well-established healthcare and it is estimated that EP accounts for ~2% of all pregnancies in Europe and North America [2]. On the contrary, in developing countries, due to poor medical and economic conditions, limited antenatal visits and prevention programs, not only it is difficult to find epidemiological data but there are important limitations in the understanding of the risk factors and management of EP [2]. The main risk factors for EP are the use of an intrauterine device at the time of conception, *Chlamydia trachomatis* and Neisseria gonorrhea infections, current or past history of pelvic inflammatory disease, previous EP, iron deficiency, and smoking cigarettes [3]. The gold standard for diagnosis is the serum concentrations of human β chorionic gonadotropin (hCG) and transvaginal ultrasound while clinical evaluation is not reliable as many women with EP report no pain nor adnexal tenderness and often it may be confused with miscarriage or induced abortion, a problem with the ovary or with a pelvic inflammatory disease [3]. In developing countries not only the gold standard is often unavailable, but women are often not aware of possible conditions and their complications and have no access to proper sexual and reproductive health services nor antenatal care [4]. Likewise, the EP management in developed countries is standardized both for stable patients, which can be treated medically with methotrexate injection, or surgically with the removal of the fallopian tube, both for unstable patients requiring emergency surgery to stop life-threatening hemorrhage [5]. In limited resources settings, instead, surgery, mostly performed by laparotomy, remains the main treatment and, due to late diagnosis, it is often performed in emergency with frequent tubal rupture and hemoperitoneum and, thus, higher risk of complications and mortality [3].

We reported a 33-year-old woman presenting a 41 weeks abdominal pregnancy successfully managed in a low-resource setting.

## Case report

A 33-year-old woman presented with a prolonged (41 weeks) pregnancy without labor and history of fourth pregnancy with three births, one stillbirth and two live children.

**Figure 1:**
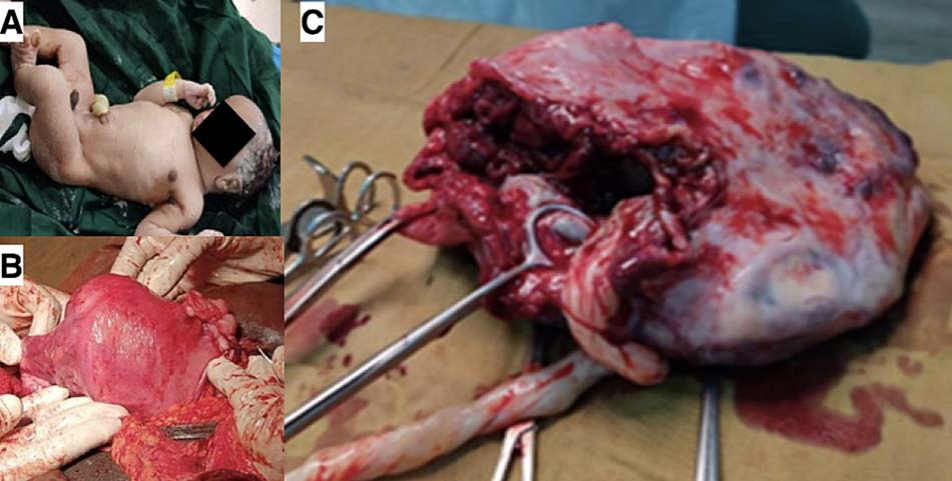
Live newborn after prolonged abdominal pregnancy (A), placenta adhesions to the left annex of the uterus (B), and adnexectomy (C).

At admission, the patient reported abdominal pain and discomfort due to fetal mobilization, with good baby movement, anorexia, and no other complaints. She presented a prenatal record of 10 consultations carried out in a rural context with no ultrasound availability and no complication. She was HIV positive on treatment with Tenofovir, Lamivudine, and Dolutegravir and tested negative for syphilis. At clinical examination, blood pressure levels were normal (115/83 mmHg), heart rate 106 bpm, respiratory rate 18 cpm, temperature 36.5°C, and cardiopulmonary auscultation unchanged. The abdomen was painful on superficial and deep palpation, the fetus was palpated in a longitudinal position, breech presentation, fundus height of 37 cm, auscultation of the fetal cardiac focus in the right hypochondrium at 130 bpm, without uterine dynamics. Upon vaginal examination, the posterior cervix was long and impervious. The ultrasound revealed a single intrauterine fetus, fetal heartbeat positive, breech presentation, biparietal diameter of 9.3 cm, femur length of 7.2 cm, and occlusive placenta previa and severe oligoamnios. Emergency cesarean section was performed. The abdominal cavity was accessed where the gestational sac was found, the empty uterus next to the gestational sac slightly increased in size. The amniotic membrane was opened and the newborn, a live male weight 2600 g was delivered with Apgar score of 6 at first minute and 8 at fifth minute (Fig. 1A). A small amount of clear amniotic fluid was observed, the placenta was inserted into the left interstitial region, with adhesions to the left annex of the uterus (Fig. 1B). Thus, the left adnexectomy was performed (Fig. 1C). Surgery was uneventful, postoperative course had no complication, and the mother and child were discharged 4 days after surgery. Importantly, 1 week follow up was regular for both.

## Discussion

EP represents a potential highly preventable and treatable condition and, especially when early detected, the chances of successful treatment are high, leading to a low risk of complications and mortality. However, these optimal conditions are characteristics of high-income countries while in undeveloped and developing countries EP remains an underestimated and underdiagnosed condition leading to urgency and fatal outcomes. The main reasons are the lack of diagnostic tools as hCG and transvaginal ultrasound and limited access to proper health care system and service. However, the higher rate of morbidity and mortality seems also related to country or region’s combined educational, economic, and medical levels reflecting a strong role of social determinants of health [3].

The successful management of this case represents a rare and extraordinary case that reflects the poor social-economic context, limited resources but also the appropriateness of the care provided in this complex case.

Considering the limited scientific literature available especially in low-income countries, further research and investigation are necessary to better understand the underlying factors contributing to EP in low-resource settings. Moreover, considering the various factors such as ethnicity, economic status, and educational levels, it is mandatory to develop effective public health policies that address these disparities and provide enhanced protection for vulnerable women. Finally, it is crucial to promote early diagnosis and treatment of EP especially in low-resource settings to mitigate its impact on women and child health.
